# New material platform for superconducting transmon qubits with coherence times exceeding 0.3 milliseconds

**DOI:** 10.1038/s41467-021-22030-5

**Published:** 2021-03-19

**Authors:** Alexander P. M. Place, Lila V. H. Rodgers, Pranav Mundada, Basil M. Smitham, Mattias Fitzpatrick, Zhaoqi Leng, Anjali Premkumar, Jacob Bryon, Andrei Vrajitoarea, Sara Sussman, Guangming Cheng, Trisha Madhavan, Harshvardhan K. Babla, Xuan Hoang Le, Youqi Gang, Berthold Jäck, András Gyenis, Nan Yao, Robert J. Cava, Nathalie P. de Leon, Andrew A. Houck

**Affiliations:** 1 Department of Electrical Engineering, Princeton University, Princeton, NJ USA; 2 Department of Physics, Princeton University, Princeton, NJ USA; 3 Princeton Institute for the Science and Technology of Materials, Princeton University, Princeton, NJ USA; 4 Department of Chemistry, Princeton University, Princeton, NJ USA

**Keywords:** Superconducting devices, Superconducting properties and materials, Quantum information, Qubits

## Abstract

The superconducting transmon qubit is a leading platform for quantum computing and quantum science. Building large, useful quantum systems based on transmon qubits will require significant improvements in qubit relaxation and coherence times, which are orders of magnitude shorter than limits imposed by bulk properties of the constituent materials. This indicates that relaxation likely originates from uncontrolled surfaces, interfaces, and contaminants. Previous efforts to improve qubit lifetimes have focused primarily on designs that minimize contributions from surfaces. However, significant improvements in the lifetime of two-dimensional transmon qubits have remained elusive for several years. Here, we fabricate two-dimensional transmon qubits that have both lifetimes and coherence times with dynamical decoupling exceeding 0.3 milliseconds by replacing niobium with tantalum in the device. We have observed increased lifetimes for seventeen devices, indicating that these material improvements are robust, paving the way for higher gate fidelities in multi-qubit processors.

## Abstract (web summary)

Quantum computers based on superconducting transmon qubits are limited by single qubit lifetimes and coherence times, which are orders of magnitude shorter than limits imposed by bulk material properties. Here, the authors fabricate two-dimensional transmon qubits with both lifetimes and coherence times longer than 0.3 milliseconds by replacing niobium with tantalum in the device.

## Introduction

Steady progress in improving gate fidelities for superconducting qubits over the last two decades has enabled key demonstrations of quantum algorithms^1–3^, quantum error correction^4–6^, and quantum supremacy^7^. These demonstrations have relied on either improving coherence through microwave engineering to avoid losses associated with surfaces and interfaces^8–10^ and to minimize the effects of thermal noise and quasiparticles^11–14^, or by realizing fast gates using tunable coupling^15,16^. By contrast, little progress has been made in addressing the microscopic source of loss and noise in the constituent materials. Specifically, the lifetime (*T*_1_) of the two-dimensional (2D) transmon qubit has not reliably improved beyond 100 μs since 2012^17,18^, and to date the longest published *T*_1_ is 114 μs^19^, consistent with other recent literature reports^20–22^.

The lifetimes of current 2D transmons are believed to be limited by microwave dielectric losses^23–25^. However, the expected loss tangent of the bulk constituent materials should allow for significantly longer lifetimes. For example, if the only source of loss is high-purity bulk sapphire with loss tangent <10^−9 ^^26,27^, *T*_1_ would exceed 30 ms. Although it is notoriously difficult to pinpoint microscopic loss mechanisms, this suggests that losses are dominated by uncontrolled defects at surfaces and interfaces, by material contaminants, or by quasiparticles trapped at the surface^28^. Here we demonstrate that a significant improvement over the state of the art in 2D transmon qubits can be achieved by using tantalum as the superconductor in the capacitor and microwave resonators, replacing the more commonly used niobium. We hypothesize that the complicated stoichiometry of oxides at the niobium surface can include non-insulating species^29–31^ that leads to additional microwave loss, and that the insulating oxide of tantalum^32,33^ reduces microwave loss in the device. We observe a time-averaged *T*_1_ exceeding 0.3 ms in our best device and an average *T*_1_ of 0.23 ms averaged across all devices, a significant improvement over the state of the art.

## Results

### Transmon fabrication and measurement

**Fig. 1:**
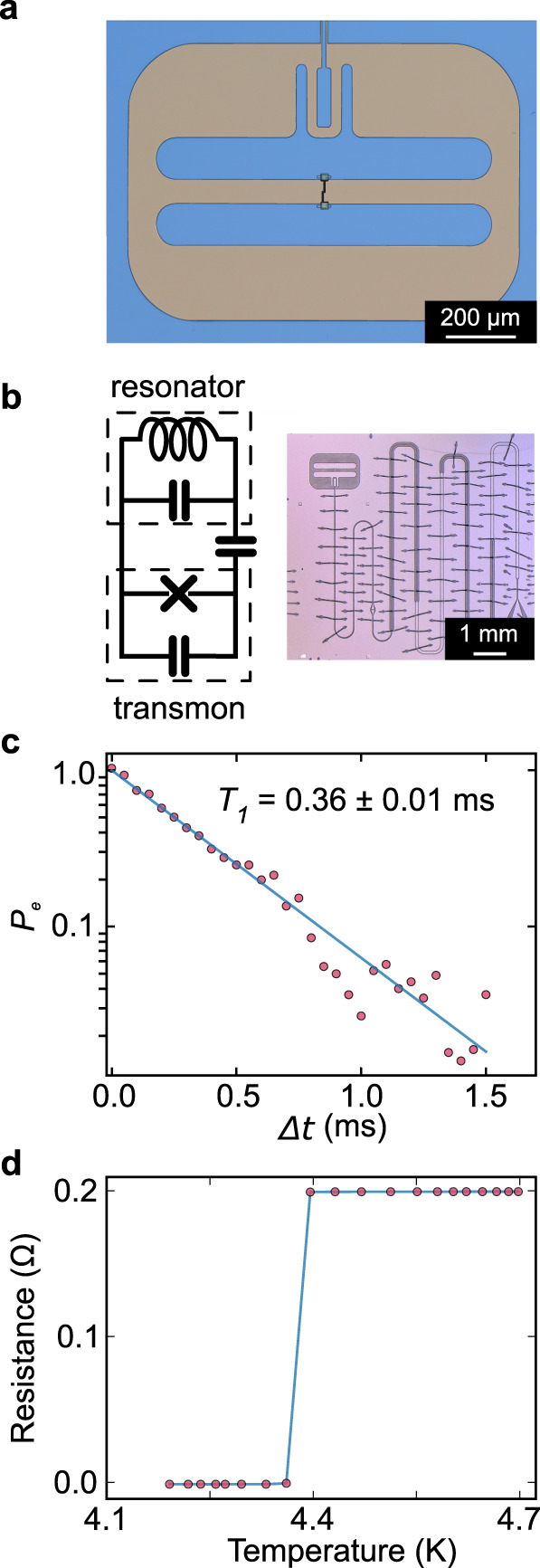
Tantalum-based transmon superconducting qubit. **a** False-colored optical microscope image of a transmon qubit. The transmon consists of a Josephson junction shunted by two large capacitor islands made of tantalum (blue) on sapphire (gray). **b** Device layout image and corresponding circuit diagram of the transmon qubit coupled to the resonator via a coupling capacitor. **c** *T*_1_ measurement of Device 18, showing the excited state population *P*_*e*_ as a function of delay time Δ*t*. Line represents a single exponential fit with a characteristic *T*_1_ time of 0.36 ± 0.01 ms. **d** Four-probe resistance measurement of the tantalum film showing *T*_*c*_ = 4.38 ± 0.02 K, consistent with the critical temperature of *α*-tantalum.

To fabricate qubits (see “Methods”), tantalum is commercially deposited on sapphire substrates by sputtering while heating the substrate to around 500 °C to ensure growth of the BCC *α* phase^34,35^. We then use photolithography and a wet chemical etch to define the capacitor and resonator of the device, followed by electron beam lithography and an in situ ion etch before electron beam evaporation of an aluminum and aluminum oxide Josephson junction (Fig. 1a). Between most key steps of the fabrication process, we use solvent and piranha cleaning to reduce contamination introduced during fabrication. The transmon is capacitively coupled to a lithographically defined resonator (Fig. 1b), allowing us to dispersively measure the state of the qubit^36^. To determine *T*_1_, we excite the qubit with a *π*-pulse and measure its decay over time at a temperature between 9 and 20 mK. In our best device, our highest *T*_1_ measurement is 0.36 ± 0.01 ms (Fig. 1c). We verify that the deposited tantalum film is in the *α* phase by measuring resistance as a function of temperature. The observed superconducting critical temperature (*T*_*c*_) is around 4.3 K, which is consistent with the intended phase (Fig. 1d) rather than the tetragonal *β* phase, which has a *T*_*c*_ below 1 K^37,38^.

**Fig. 2:**
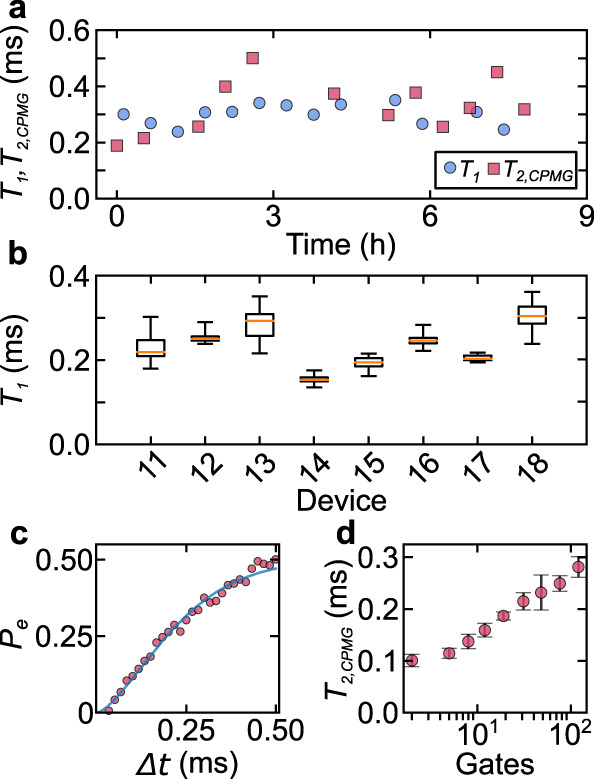
Lifetime and decoherence measurements. **a** Lifetime (*T*_1_) and coherence time with dynamical decoupling (*T*_2,*CPMG*_) of Device 18 over time. **b** Summary of *T*_1_ time series measurements of all devices fabricated with a wet etch and piranha cleaning steps. Details about the specific processing steps for each device are given in Supplementary Table 1 and “Methods.” The yellow line shows the median, while the box spans the middle two quartiles of the data. The whiskers show the extremal measurements. Data for Devices 13, 14, and 17 are the average of 19, 14, and 7 individual *T*_1_ measurements, respectively, while the rest are the average of at least 32 measurements. Each device was measured over a period of hours to days, and Devices 11 and 13 include data from multiple dilution refrigerator cycles. **c** *T*_2,Echo_ measurement of Device 18, showing the excited state population *P*_*e*_ as a function of delay time Δ*t*. Solid blue line shows a stretched exponential fit to the data. The fit gives *T*_2,Echo_ = 249 ± 4 μs. **d** *T*_2,CPMG_ of Device 11 as a function of the number of gates in a CPMG pulse sequence. The error bars denote one standard deviation in the data.

We observe reproducible, robust enhancement of *T*_1_ across all devices fabricated with this process. The lifetime of a given qubit fluctuates over time, with a standard deviation of around 7% of the mean (Fig. 2a). Results for eight devices are presented in Fig. 2b, with the time-averaged *T*_1_ ranging from 0.15 to 0.30 ms, and an average *T*_1_ of 0.23 ms across all devices, qualitatively exceeding the *T*_1_ of prior 2D transmon devices. We note that the highest observed *T*_1_ and *T*_2_ are achieved in a device with a thinner aluminum layer (Device 18, see “Methods”). The time-averaged coherence time, *T*_2,Echo_, in our best device is 0.20 ± 0.03 ms (a trace is shown in Fig. 2c). We can extend the coherence time using a Carr–Purcell–Meiboom–Gill (CPMG) pulse sequence^39^ (Fig. 2d), and we achieve a time-averaged *T*_2,CPMG_ of 0.38 ± 0.11 ms in our best device (Fig. 2a). The spectral noise density extracted from dynamical decoupling measurements is consistent with 1/*f* noise (Supplementary Fig. 11) suggesting that this noise can be mitigated with a Floquet drive^40^. We note that there are small variations in processing and packaging between these eight devices, which are outlined in “Methods” and Supplementary Table 1.

### Device iterations

In addition to the eight qubits presented in Fig. 2, we present data on a total of 21 transmon qubits that were fabricated using different geometries, materials, and fabrication processes in Supplementary Table 1. We note that switching from niobium to tantalum alone increased the average *T*_1_ for a Purcell-filtered qubit to 150 μs (Device 2), already a significant improvement over the best published 2D transmon lifetime. To study the impact of heating the substrate during deposition, we made a device from niobium sputtered at 500 °C (Device Nb2). This resulted in a *T*_1_ of 79 ± 1 μs, an improvement over our previous niobium devices, but qualitatively lower than tantalum-based devices. This indicates that thermal cleaning of the substrate may play a role in enhancing *T*_1_, but does not completely explain our improved coherence.

Iterative improvements to processing, including the use of wet etching to pattern the tantalum layer and the introduction of additional cleaning steps, further improved qubit lifetimes to the levels reported in Fig. 2 (Devices 11–18). Specifically, a piranha cleaning process (see “Methods”) was introduced to clean particulates and contaminants from the substrate and metal surfaces. Removal of particulates was verified using atomic force microscopy (AFM), and the signal due to adventitious carbon measured by x-ray photoelectron spectroscopy (XPS) is attenuated after cleaning (Supplementary Fig. 3). In addition, we find that the introduction of an optimized wet etch process to pattern the tantalum resulted in improved edge morphology compared with reactive-ion etching (Supplementary Fig. 2). Of the ten devices measured prior to the optimized wet etch, none had a *T*_1_ in excess of 200 μs; of the eight patterned with the optimized wet etch and fabricated with our cleaning procedure, six had a *T*_1_ > 200 μs. These observations imply that residue and poor edge and surface morphology may limit qubit lifetimes for our tantalum devices.

### Characterization of tantalum films

**Fig. 3:**
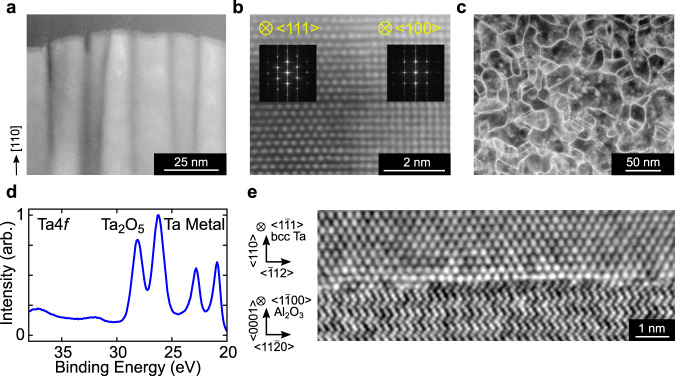
Microscopy and spectroscopy of tantalum films. **a** STEM image of the tantalum film, showing single-crystal columns with the growth direction oriented along the [110] axis. **b** Atomic-resolution STEM image of an interface between two columns, viewed from $\left\langle 1\bar{1}1\right\rangle$ and $\left\langle 001\right\rangle$ zone axes, respectively. Fourier transforms (insets) of the image show that the columns are oriented with the image plane perpendicular to the $\left\langle 111\right\rangle$ or $\left\langle 100\right\rangle$ directions. **c** STEM image of a horizontal device cross section, showing grain boundaries. Image contrast at grain boundaries results from diffraction contrast caused by interfacial defects. **d** XPS spectrum of a device, exhibiting peaks from tantalum metal and Ta_2_O_5_. Other oxidation states of tantalum are expected to have binding energies between 22.2 and 23.8 eV^42, 43^. **e** High-resolution STEM with integrated differential phase contrast imaging of the interface between the sapphire and tantalum showing epitaxial growth.

Because thin film structure has been observed to affect qubit performance for niobium-based qubits^31^, and because the crystal structure of thin tantalum films sensitively depends on deposition parameters^34,37^, we present detailed characterization of the deposited tantalum films. Scanning transmission electron microscopy (STEM) of a film cross section reveals a columnar structure, with the growth direction oriented along the [110] axis (Fig. 3a) and these observations are corroborated by probing a larger area with x-ray diffraction measurements (Supplementary Fig. 7). Atomic-resolution STEM confirms the BCC structure of the film and reveals that the individual columnar grains are single-crystal, with the front growth face perpendicular to either the $\left\langle 100\right\rangle$ or $\left\langle 111\right\rangle$ directions (Fig. 3b). The different orientations result from the underlying three-fold symmetry of the sapphire crystal structure about its c-axis^41^. A top-down plane view cross-sectional STEM shows that the grains range in size from around 5–50 nm (Fig. 3c). We study the tantalum oxide on our devices using XPS, which shows two sets of spin-orbit split doublet peaks with binding energy between 20 and 30 eV associated with 4*f* core ionization of Ta metal (lower binding energy) and Ta_2_O_5_ (higher binding energy) (Fig. 3d)^42,43^. The relative intensity of the metal and oxide peaks indicates that the oxide is ~2-nm thick (see Supplementary Information), consistent with angle-resolved XPS and high-resolution STEM measurements (Supplementary Fig. 9). Last, we directly image the interface between the sapphire surface and the sputtered tantalum using integrated differential phase contrast imaging under STEM (Fig. 3e). The interface shows an atomically sharp boundary with clear evidence of epitaxial growth, in which the tantalum atomic layer is directly grown on top of the oxygen atomic layer in the sapphire.

## Discussion

We have demonstrated that tantalum 2D transmon qubits exhibit longer *T*_1_ and *T*_2_ than the previous state of the art with remarkable consistency. Building on these relatively simple materials improvements, there are several areas of future research. First, *T*_2,Echo_ is shorter than *T*_1_ for all tantalum devices measured. Better shielding^44^ and filtering^45^ of tantalum transmons may enable measurements with unprecedentedly long *T*_2_, allowing for the exploration of microscopic mechanisms of relaxation and decoherence. In addition, much can be learned from more systematic characterization of the effects of specific material properties on microwave losses. In particular, there are many open questions about the relative importance of oxide properties on device performance. An exciting avenue is to explore the detailed scaling of *T*_1_ with surface participation by measuring tantalum qubits with different geometries. In addition, we are exploring the impact of tantalum grain size and heteroepitaxial growth interface quality on *T*_1_ and *T*_2_. Furthermore, it has been well-established that multi-qubit devices suffer from significant variation between qubits^21^, as well as variation over time in the same qubit^46^. An interesting question is how particular material choices quantitatively affect these variations, and whether judicious material choice can narrow the distribution of device properties. Finally, we note that while we have not made a direct comparison to all-aluminum qubits in this paper, the average *T*_1_ achieved here is better than twice as long as the best published all-aluminum 2D transmon^22,47^. Further, our process produces a higher and more consistent average *T*_1_ than the reported state of the art in 3D all-aluminum transmons^48–50^. The feasibility of eliminating aluminum by making all-tantalum qubits has yet to be explored.

More broadly, our results demonstrate that systematic material improvements are a powerful approach for rapid progress in improving quantum devices. We have recently employed similar targeted material techniques to improve spin coherence of shallow nitrogen vacancy centers in diamond^51^, and we note that many other quantum platforms are also limited by noise and loss at surfaces and interfaces, including trapped ions^52,53^, shallow donors^54,55^, and semiconductor quantum dots^56^. Our general approach of modifying and characterizing the constituent materials may allow for directed, rational improvements in these broad classes of systems as well.

## Methods

### Tantalum deposition and lithography

The 2D transmon qubits are fabricated on c-plane sapphire substrates (Crystec GmbH) that are 0.53-mm thick and double-side polished (Supplementary Fig. 1). Prior to deposition, the wafer is cleaned by the commercial deposition company, Star Cryoelectronics, by dipping in a piranha solution (H_2_SO_4_ and H_2_O_2_) then cleaning with an oxygen plasma (Technics PE-IIA System) immediately before loading into the sputterer.

Tantalum is deposited on the sapphire substrate at high temperature (Star Cryoelectronics, alpha tantalum, 500 °C deposition, 200-nm thickness, no seed layer, sapphire preclean with piranha and oxygen plasma). Before photolithography, the tantalum-coated substrates are placed in a 2:1 mixture of H_2_SO_4_ and H_2_O_2_ for 20 min (hereafter “piranha” refers to this specific chemical ratio and time duration) then heated on a hotplate for 5 min at 140 °C before AZ 1518 resist is spun (Merck KGaA). The resist is patterned using a direct-write process (2 mm write head on a Heidelberg DWL 66+ Laser Writer). After developing (85 s in AZ 300MIF developer from Merck KGaA), the resist is hard-baked for 2 min at 115 °C. Unwanted residual resist is removed using a gentle oxygen descum (2 min in 30-mTorr O_2_ with 20 W/200 W RF/ICP coil power in a Plasma-Therm Apex SLR). Next, the tantalum is etched in a 1:1:1 ratio of HF:HNO_3_:H_2_O (Tantalum Etchant 111 from Transene Company, Inc.) for 21 s. After stripping resist, the device is solvent-cleaned by sonicating in sequential baths of toluene, acetone, methanol, and isopropyl alcohol for ~2 min each ("TAMI-cleaned”) then piranha-cleaned. The patterned tantalum is prepared for electron beam lithography to define Josephson junctions (resists MMA 8.5 MAA and 950 PMMA, with a 40-nm layer of evaporated aluminum to dissipate charge), then the chips are diced into 7 × 7-mm squares.

Liftoff patterns for Manhattan junctions^57^ with overlap areas of ~0.03 μm^2^ are then exposed (Elionix ELS-F125). The anticharge layer is removed through a 4-min bath in MF 319 (Rohm and Haas Electronic Materials LLC) followed by a 50-s bath in a 1:3 mixture of methyl isobutyl ketone to isopropyl alcohol. Next, the device is loaded into a Plassys MEB 550S electron beam evaporator and ion-milled (400 V, 30 s along each trench of the junction). Immediately after, 15 nm of aluminum is deposited at 0.4 nm/s at a pressure of ~10^−7^ mBar, followed by a 15-min, 200-mBar oxidation period. Finally, 54 nm of aluminum is deposited to form the second layer of the junction, with the same evaporation parameters (for Device 18, 15 and 19 nm of aluminum are deposited, respectively). The resist is then removed by soaking the sample in Remover PG (Kayaku Advanced Materials, Inc.) for ~3 h at 80 °C, briefly sonicating in hot Remover PG, then swirling in isopropyl alcohol.

### Device packaging

The completed devices are first mounted to a printed circuit board (PCB). The edge of the tantalum ground plane is firmly pressed against the PCB’s copper backside, sandwiched between the PCB and a piece of aluminum-coated oxygen-free copper (Supplementary Fig. 5b). The device is then wirebonded (Supplementary Fig. 5a, d). An aluminum-coated oxygen-free copper lid is sometimes placed above the qubit (Supplementary Table 1 column “Enclosure Lid Removed”), forming a superconducting enclosure partially surrounding the qubit. The device is mounted in a dilution refrigerator with a base temperature of ~9–20 mK. The qubit and PCB are wrapped in several layers of aluminized mylar sheeting and suspended by an oxygen-free copper rod in the middle of an aluminum cylinder coated with microwave-attenuating epoxy or sheeting (Laird Performance Materials Eccosorb Cr or Loctite Stycast). This cylinder is enclosed in a mu-metal can to reduce the penetration of ambient magnetic fields into the aluminum during the superconducting transition. Both cans are then wrapped in several layers of mylar sheeting.

We note that all of the double-pad transmons presented in this text are positioned ~2 mm away from the copper traces on the PCB (Supplementary Fig. 5a), which could result in loss due to parasitic coupling of the qubit to the resistive traces. In order to reduce this possible source of loss, devices fabricated with the single-pad geometry were moved close to the center of the sapphire chip (Supplementary Fig. 5d).

### Measurement setup

Each transmon is capacitively coupled to a microwave resonator, allowing the state of the qubit to be measured dispersively^36^. The transmon frequencies range from 3.1 to 5.5 GHz while the resonators range in frequency from 6.8 to 7.3 GHz. An overview of the setup used to measure a majority of the devices is given in Supplementary Fig. 6. An Agilent E8267D vector signal generator, Holzworth HS9004A RF synthesizer, and Keysight M9330A arbitrary waveform generator are used to synthesize the excitation and measurement pulses. The input signals are combined into a single line and then attenuated on each plate of the dilution refrigerator. An additional filter made of Eccosorb CR110 epoxy is placed in the aluminum can to attenuate high-frequency radiation. Measured in reflection, the output signal is sent through a circulator (Raditek RADC-4-8-cryo-0.01-4K-S23-1WR-ss-Cu-b), two isolators (Quinstar QCI -075900XM00), superconducting wires, and then a high-electron-mobility transistor amplifier (Low Noise Factory LNF-LNC4_8C) at 4 K. After the signal is amplified at room temperature (through two MITEQ AFS4-00101200 18-10P-4 amplifiers), it is measured in a homodyne setup by first mixing it with a local oscillator (Holzworth HS9004A), further amplifying (Stanford Research Systems SR445a), and then digitizing (Acqiris U1084A).

### Tantalum etch

Initially we etched tantalum using a reactive-ion etch (8:3:2 CHF_3_:SF_6_:Ar chemistry at 50 mTorr, RF/ICP power of 100/100 W). However, scanning electron microscopy (SEM) images showed that reactive-ion etches can produce rough edges as well as small pillars and boulders near the sidewalls, likely due to micromasking (Supplementary Fig. 2a, b). The anomalous objects in Supplementary Fig. 2b remained after the device was cleaned in piranha solution and treated in an oxygen plasma. In order to avoid these fabrication problems, we employed a wet etch composed of 1:1:1 HF:HNO_3_:H_2_O. We found that several resists delaminated before the tantalum was etched through, leaving the sidewalls and nearby tantalum visibly rough in SEM (Supplementary Fig. 2c). This problem was circumvented by using thick AZ 1518 resist (~2-μm tall), which left cleaner sidewalls (Supplementary Fig. 2d). Comparing Devices 4–10 with Devices 11–18 in Supplementary Table 1, we note that the optimized wet etch likely improved *T*_1_.

### Sapphire preparation

During recipe development, we aggressively cleaned and etched some wafers before tantalum was deposited. After dicing the resist-covered sapphire wafers, stripping the resist, and sonicating in solvents we found surface contamination. In particular, AFM revealed an abundance of particulates (Supplementary Fig. 3a) which were removed by cleaning in piranha solution (Supplementary Fig. 3b). The etched and piranha-cleaned surface was smooth (R_*a*_ of 80 pm) and did not show any signs of roughening (Supplementary Fig. 3b). In addition, the carbon signal in XPS was attenuated by a factor of 5 after piranha cleaning, illustrating a reduction in carbon contamination (Supplementary Fig. 3c). XPS also revealed zinc contamination that persisted through a piranha clean, but was removed by etching the sapphire substrate in heated sulfuric acid (Supplementary Fig. 3d). We note that sapphire wafers that had not been diced, stripped of resist, and sonicated in solvents displayed few particulates in AFM, and XPS showed a minimal carbon signal and no detectable zinc signal.

We prepare the sapphire surface using this sulfuric acid etch in Devices 9–14 and 17. In these devices, the wafers are covered with a protective layer of photoresist and then diced into 1 inch squares. After removing resist, the squares are TAMI-cleaned and piranha-cleaned. Next, the sapphire is placed into a quartz beaker filled with H_2_SO_4_ sitting on a room temperature hotplate. The hotplate is set to 150 °C for 20 min, followed by a 10-min cooldown period before removing the device. We estimate that <1 nm of the surface is removed through this procedure^58^. To avoid residue from the etch, the device is piranha-cleaned again. The device is then packaged, shipped, and loaded into a sputterer without further cleaning.

Calibrating the time and temperature of the sapphire etch is critical to maintaining a smooth surface morphology while still removing zinc. In particular, polycrystalline aluminum sulfates form on the sapphire surface after heating in sulfuric acid for too long and at too high of a temperature (Supplementary Fig. 4a)^58^. We developed our sapphire etch recipe by (1) looking for crystal formation in an optical microscope, (2) ensuring that zinc was removed in XPS, and (3) checking that we preserved smooth surface morphology in AFM. We note that the zinc appeared to be inhomogeneously distributed on the surface and so we routinely checked multiple spots in XPS. After adjusting the time and temperature to the optimum procedure outlined above, we did not detect any crystal formation.

In addition, we observed surface contamination with AFM from etching sapphire in borosilicate glassware. An example of surface particulate contamination is shown in Supplementary Fig. 4b. Switching to a quartz beaker solved this issue.

We note that Devices 16 and 18 were not processed using the sapphire etch, and they exhibited *T*_1_ over 0.2 ms. In the future, we are interested in studying the impact of sapphire material properties on device performance. We plan to fabricate devices on higher-purity sapphire, remove polishing-induced strain by etching more of the substrate, and anneal to form an atomically smooth surface^58^.

### Fabrication and packaging procedure iterations

Supplementary Table 1 summarizes different iterations of the fabrication procedure. Initially we made a tantalum transmon using our standard niobium processing techniques (reactive-ion etching, no acid cleaning). This material switch alone improved the coherence time by more than a factor of four compared to the control sample (Supplementary Table 1, Devices 1a and Nb1). We then began to iterate our packaging and fabrication techniques to explore the new dominant loss mechanisms.

First we minimized losses unrelated to the qubit materials and interfaces. We reduced the density of photonic states at the qubit frequency by means of a Purcell filter (Device 2a and all subsequent devices)^59^. We also deposited aluminum shielding on a majority of the copper enclosure immediately surrounding the device to reduce dissipative currents induced by the qubit in the surrounding metal. At the same time, we introduced a mylar sheet wrapped around the PCB as an extra layer of shielding. Both added layers give additional protection from high-energy radiation (Device 2b and all subsequent devices).

Next we focused on reducing material contaminants. XPS measurements revealed significant carbon residue that persisted after solvent-based cleaning. Accordingly, we reduced carbon contamination by adding a piranha clean before spinning e-beam resist (Device 4 and all subsequent devices). As mentioned above, we also cleaned the sapphire substrate prior to tantalum deposition. For Devices 1–8, 15–16, and 18 as well as Nb1 and Nb2, the sapphire substrate was dipped in a piranha solution and cleaned with an oxygen plasma (Technics PE-IIA System) immediately before loading into the sputterer. For the rest of the sapphire devices, we cleaned the substrate with the sapphire etch described above (Supplementary Note 2), packaged and shipped the samples, then deposited the tantalum.

We then focused on the tantalum etch, described in more detail above. Devices 1–6, Nb1–2, and Si1 were all fabricated with reactive-ion etching. Devices 7–10 were made using initial versions of the wet etch (using different resists, etch times, and acid concentrations), where the etch clearly roughened the sidewalls (Supplementary Fig. 2c). Devices 11–18 were made using the optimized wet etch.

## Supplementary information

Supplementary Information

## Data Availability

The data are available upon reasonable request.

## Code availability

The codes are available upon reasonable request.
