# Supplementary material for: New material platform for superconducting transmon qubits with coherence times exceeding 0.3 milliseconds
Source: Nat Commun. 2021 Mar 19;12:1779. doi: 10.1038/s41467-021-22030-5 (PMC7979772; doi:10.1038/s41467-021-22030-5)
Supplement: Supplementary file 1 — Supplementary Information [file 41467_2021_22030_MOESM1_ESM.pdf]

Supplementary Materials for
New material platform for superconducting transmon qubits with coherence times exceeding 0.3 milliseconds

Alexander P. M. Place[1†], Lila V. H. Rodgers[1†], Pranav Mundada[1], Basil M. Smitham[1], Mattias Fitzpatrick[1], Zhaoqi Leng[2], Anjali Premkumar[1], Jacob Bryon[1], Andrei Vrajitoarea[1], Sara Sussman[2], Guangming Cheng[3], Trisha Madhavan[1], Harshvardhan K. Babla[1], Xuan Hoang Le[1], Youqi Gang[1], Berthold Jäck[2], András Gyenis[1], Nan Yao[3], Robert J. Cava[4], Nathalie P. de Leon[1], Andrew A. Houck[1*]

[†]These authors contributed equally to this work.

[*]To whom correspondence should be addressed; E-mail: aahouck@princeton.edu.

February 1, 2021

Supplementary Note 1: Device Geometry

We measured two different types of transmons: devices with double-pad capacitors, where neither pad has a direct ground connection (Fig. 1a), and single-pad devices[1], where the ground plane serves as one side of the transmon's capacitor (Supplementary Fig. 5d). Two different geometries of double-pad devices were tested on sapphire: Devices 1-6 had a 60 μm gap between capacitor pads, while Devices 7-12 and 15-18 had a 70 μm gap and a smaller charging energy.

Cavity and Purcell Filter

The coupling capacitor design between the resonator and Purcell filter was consistent across all devices on sapphire. The cavity linewidth of Device 18 was 380 $\pm$ 2 kHz.

To limit Purcell decay we capacitively couple the readout resonator to a stepped impedance filter[2]. The filter is composed of two repetitions of two alternating sections with impedances of 26 Ω and 127 Ω with lengths 5.5 mm and 8 mm, respectively.

Participation Ratio

To isolate geometric contributions to relaxation we simulated the participation ratios of the 70 μm gap double-pad geometry using a method similar to Wang et al.[3], assuming the same simplified junction geometry. A device with a dielectric layer of thickness 3 nm and dielectric constant $\epsilon = 10$, similar to the aluminum oxide layer simulated in[3], gave a substrate-metal interface participation ratio of $1.6 * 10^{-4}$, excluding the areas within 1 μm of the junction.

Supplementary Note 2: Transmon on a Silicon Substrate

We fabricated a 2D, double-pad, tantalum transmon on silicon (Device Si1) with a similar design to that used for the devices on sapphire. The primary elements that changed during the fabrication process were: (i) a different plasma etch time to avoid overetching into the silicon, (ii) no aluminum layer was deposited on top of the e-beam resist prior to e-beam lithography, and (iii) the e-beam intensity was adjusted during the lithography step. We found that reactive-ion etching severely roughened the silicon surface (17 nm RMS surface roughness, measured with a Keyence Optical Profilometer). We plan to optimize this fabrication process in the future.

Supplementary Note 3: Additional Materials Characterization

X-ray Diffraction

We use XRD to study the crystal structure of our films over a much larger area than is feasible with STEM images (Supplementary Fig. 7). An acquired spectrum of a film exhibits a strong peak corresponding to α-tantalum [110][4], corroborating STEM images that suggest that our films grow uniformly along that direction (Fig. 3a). Additionally, we observe peaks corresponding to sapphire [006][5] and α-tantalum [220][4]. We do not detect a β-tantalum [002] peak at 33.7° (2θ) (Supplementary Fig. 7, inset left)[4]. This provides further evidence along with our T_c and STEM measurements that the tantalum films are uniformly in the α phase. We note that there are a few unassigned small peaks which could result from contamination, instrumental artifacts, or impurities or defects in the tantalum films (Supplementary Fig. 7, inset right).

Grain Boundaries

We further interrogate the grain boundaries visible in a plane-view image (Supplementary Fig. 8a) by using energy dispersive x-ray spectroscopy (EDS) to perform spatially-resolved elemental analysis. We find a uniform distribution of tantalum (Supplementary Fig. 8b) and oxygen (Supplementary Fig. 8c) over the region, and no oxygen enrichment at the grain boundaries. This suggests that our films do not grow oxide between the grains, and that the image contrast observed in Supplementary Fig. 8a arises instead from diffraction contrast caused by interfacial defects.

A high-resolution STEM image of a grain boundary elucidates the crystal structure at the boundaries (Supplementary Fig. 8d). Taking a diffraction pattern of a grain boundary region indicated by a green square in Supplementary Fig. 8d gives a pattern consistent with twinning (Supplementary Fig. 8e). A diffraction pattern of the whole region in Supplementary Fig. 8d illustrates the rotational symmetries of the grains (Supplementary Fig. 8f).

Tantalum Oxide

An atomic-resolution STEM image of a 50 nm region of the tantalum surface reveals an amorphous oxide that is 2-3 nm thick (Supplementary Fig. 9a). We further study this oxide using XPS to estimate oxide thickness and composition over a larger area (250 μm spot size) (Supplementary Fig. 9b, d-f). XPS scans of the tantalum film show two sharp lower binding energy peaks assigned to tantalum metal $4f_{7/2}$ and $4f_{5/2}$ orbitals (lower binding energy to higher binding energy, respectively), two peaks at higher binding energy corresponding to the same orbitals of Ta_2O_5[6,7], and two small $5p_{3/2}$ peaks corresponding to the metal and oxide, respectively[8]. Assuming the mean free path of electrons in tantalum is 2 nm at 1480 eV[9], and only taking into account inelastic scattering, a thickness can be estimated by comparing the ratio of oxide to

metal peak areas. We corroborate this estimation using angle-resolved XPS (ARXPS), where we vary the angle between sample and detector, changing the relative distances that the emitted photoelectrons travel through the metal and oxide layers to reach the detector (Supplementary Fig. 9b). We account for this geometry in our modeling, and extract the oxide thickness at different angles (Supplementary Fig. 9c). The thickness estimation remains fairly consistent until higher angles, when other effects related to surface morphology or elastic scattering become more significant (Supplementary Fig. 9c)[10].

To investigate the variability of oxide thickness between devices, we show normal incidence XPS data from three devices from different tantalum depositions with different surface cleaning fabrication procedures (Supplementary Fig. 9d-f). In addition to variations in other fabrication steps, we note that the device shown in Supplementary Fig. 9d was only solvent cleaned, and the devices in Supplementary Fig. 9e and Supplementary Fig. 9f were piranha cleaned. The peak shapes and ratio of oxide to metal peak area are similar between all these devices, suggesting the oxide thickness and composition is robust to processing steps.

Sapphire-Tantalum Interface

We study the heteroepitaxial growth interface in our devices by directly imaging small regions of the sapphire-tantalum interface using iDPC STEM. In addition to the iDPC STEM image shown in Fig. 3e, we include an image showing the interface between sapphire and tantalum viewed from $\langle 1\bar{1}00 \rangle$ sapphire and $\langle 100 \rangle$ tantalum zone axes (Supplementary Fig. 10a). We also propose atomistic models for an ideal sapphire-tantalum interface shown in Supplementary Figures 10b and c to help visualize the lattice matching between sapphire and tantalum, and as a starting point for future studies on the impact of sapphire surface morphology on heteroepitaxial growth. For example, the interfacial dislocations visible in Fig. 3e likely result from the 12.6$\%$ lattice mismatch between the $[\bar{1}12]$ axis of tantalum and the $[11\bar{2}0]$ axis of sapphire (Supplementary Fig. 10c), as well as atomic layer steps in the sapphire that are evident in the STEM image.

XPS, AFM, XRD characterization

All XPS, AFM, and XRD data were acquired using tools in the Imaging and Analysis Center at Princeton University.

XPS was performed using a Thermo Fisher K-Alpha and X-Ray Spectrometer tool with a 250 μm spot size. The data shown in Fig. 3d, Supplementary Fig. 3c and d, and Supplementary Fig. 9d-f were obtained by collecting photoelectrons at normal incidence between sample and detector. The angle-resolved XPS (ARXPS) spectra shown in Supplementary Fig. 9b were collected by changing the angle between sample and detector. All AFM images were taken with a Bruker Dimension Icon3 tool operating in tapping mode (AFM tip from Oxford Instruments Asylum Research, part number AC160TS-R3, resonance frequency 300 kHz). The XRD spectrum shown in Supplementary Fig. 6 was collected with a Bruker D8 Discover X-Ray

Diffractometer configured with Bragg-Brentano optics. Two 0.6 mm slits were inserted before the sample, and a 0.1 mm slit was placed before the detector.

Electron Microscopy Characterization

SEM and STEM images were also collected at the Imaging and Analysis Center at Princeton University. STEM thin lamellae (thickness: 70-1300 nm) were prepared by focused ion beam cutting via a FEI Helios NanoLab 600 dual beam system (FIB/SEM). All the thin samples for experiments were polished by a 2 keV Ga ion beam to minimize the surface damage caused by the high-energy ion beam. Conventional STEM imaging, iDPC, atomic-resolution HAADF-STEM imaging and atomic-level EDS mapping were performed on a double Cs-corrected Titan Cubed Themis 300 STEM equipped with an X-FEG source operated at 300 kV and a super-X energy dispersive spectrometry (super-X EDS) system.

Lithography and etching process development SEM images were collected with a FEI Verios 460XHR SEM and a FEI Quanta 200 Environmental SEM. Various tilt angles, working distances, and chamber pressures were used to eliminate charging effects.

Supplementary Note 4: CPMG

To reduce our devices' low-frequency noise sensitivity we applied a sequence of π-pulses[11]. Each pulse had a Gaussian envelope with σ around 20-50 ns and was truncated at $\pm 2\sigma$. Due to the large number of sequential pulses, we found that reducing gate error through frequent calibration was important.

To derive the qubit's noise spectral density (Supplementary Fig. 11) from such a pulse sequence, we follow the procedure in[11]. The signal-to-noise ratio decreases as the overall delay time between initial excitation and measurement increases. For clarity, we include only delays spanning up to approximately T_1. For simplicity we assume the gates are instantaneous. We find a noise power spectral density that is well fit by $A/f^{\alpha} + B$ with α = 0.7.

Supplementary Note 5: Fitting Procedure

We fit our transmon T_1 data to $f(\Delta t) = e^{-\Delta t/T_1}$, where T_1 is a fit parameter and the function represents the population in the excited state. We fit any T_2 data taken with fringes to the fit $f(\Delta t) = 0.5e^{-\Delta t/T_{2R}} \cos(2\pi\Delta t\delta + \phi_0) + 0.5$ where T_{2R}, δ, and ϕ_0 are fit parameters. For echo and CPMG experiments, we fit our T_2 data with a stretched exponential, $f(\Delta t) = 0.5e^{-(\Delta t/T_2)^n} + 0.5$, where T_2 and n are fit parameters. If $n < 1$, the data is refit to a pure exponential. Supplementary Fig. 12 shows a representative decay for a low, average, and high value of $T_{2,CPMG}$ for the data shown in Fig. 2a. In time sequences, data traces with obvious abnormalities or poor fits as measured by root-mean-square error are discarded.

Device	Average T_1 (μs)	Max T_1 (μs)	Q (millions)	Average T_{2R} (μs)	Average T_{2E} (μs)	Average T_{2CPMG} (μs)	Purcell Filter	Al Coated Enclosure	Mylar Shielding	Piranha Clean	Sapphire Etch	Optimized Wet Etch	Enclosure Lid Removed	Thin Al Layer	Geometry
1a*	$98 \pm 2^{\dagger}$	99	1.9	-	-	-									Double Pad
1b*	$127 \pm 4^{\dagger}$	130	2.5	-	-	-									Double Pad
2a	$150 \pm 2^{\dagger}$	151	4.2	-	-	-	x								Double Pad
2b	157 ± 19	179	4.4	98 ± 8	153 ± 12	-	x	x	x						Double Pad
2c	147 ± 15	181	4.1	-	-	-	x	x	x						Double Pad
2d*	171 ± 26	223	4.8	-	-	-	x	x	x						Double Pad
3	$146 \pm 2^{\dagger}$	151	3.6	-	-	-	x	x	x						Double Pad
4	200 ± 11	219	5.3	40 ± 4	44 ± 7	-	x	x	x	x					Double Pad
5	146 ± 9	169	4.4	47 ± 5	76 ± 7	-	x	x	x	x					Double Pad
6	$58 \pm 2^{\dagger}$	58	1.8	-	-	-	x	x	x	x					Double Pad
7a	$166 \pm 1^{\dagger}$	176	5.4	$18 \pm 1^{\dagger}$	$49 \pm 1^{\dagger}$	-	x	x	x	x					Double Pad
7b*	184 ± 18	220	6.1	-	-	-	x	x	x	x					Double Pad
8	$135 \pm 1^{\dagger}$	135	4.5	-	-	-	x	x	x	x					Double Pad
9	$127 \pm 1^{\dagger}$	133	3.0	-	-	-	x	x	x	x	x				Double Pad
10	$139 \pm 3^{\dagger}$	139	3.9	-	-	-	x	x	x	x	x				Double Pad
11a	212 ± 11	255	5.2	41 ± 1	53 ± 4	-	x	x	x	x	x	x			Double Pad
11b	273 ± 27	303	6.7	101 ± 5	144 ± 9	-	x	x	x	x	x	x	x		Double Pad
11c	257 ± 19	301	6.2	68 ± 6	87 ± 9	281 ± 20	x	x	x	x	x	x	x		Double Pad
12	253 ± 11	290	6.0	53 ± 2	71 ± 2	-	x	x	x	x	x	x			Double Pad
13a	288 ± 35	351	6.6	$68 \pm 6^{\dagger}$	-	-	x	x	x	x	x	x			Single Pad
13b*	$247 \pm 22^{\dagger}$	277	5.6	-	-	-	x	x	x	x	x	x			Single Pad
14*	154 ± 9	175	4.0	-	$18 \pm 1^{\dagger}$	-	x	x	x	x	x	x			Single Pad
15	194 ± 13	215	4.6	-	123 ± 7	-	x	x	x	x		x	x		Double Pad
16	247 ± 14	283	5.5	-	62 ± 2	$122 \pm 4^{\dagger}$	x	x	x	x		x	x		Double Pad
17	205 ± 8	217	5.0	$58 \pm 1^{\dagger}$	78 ± 4	$93 \pm 6^{\dagger}$	x	x	x	x	x	x	x		Double Pad
18	303 ± 32	362	7.1	$105 \pm 3^{\dagger}$	201 ± 28	377 ± 111	x	x	x	x		x	x	x	Double Pad
Nb1*	$23 \pm 1^{\dagger}$	23	0.4	-	$42 \pm 1^{\dagger}$	-									Double Pad
Nb2	$79 \pm 1^{\dagger}$	82	2.0	-	-	-	x	x	x						Double Pad
Si1	$118 \pm 2^{\dagger}$	124	2.3	$44 \pm 2^{\dagger}$	-	-	x	x	x						Double Pad

Supplementary Table 1. Summary of devices. Here we include measurements of devices with different designs, fabrication procedures, and packaging. Devices labeled "Nb" were made with niobium instead of tantalum (Nb1 was heated to 350°C then cooled for 20 minutes before deposition, Nb2 was deposited at approximately 500°C) and all other devices were made from tantalum. Device Si1 was composed of about 200 nm of tantalum deposited on high-resistivity silicon. Each individual device is labeled with its own number. Devices marked with an additional letter indicate different thermal cycles of the same device. Entries marked with a "†" had three or fewer repeated measurements, and the reported errors were calculated by propagating the fit uncertainties. Otherwise the errors were calculated by finding the standard deviation of multiple measurements. Devices labeled with a "$*$" were fit without constraining the line of best fit to be normalized and have the proper offset. The average $T_{2,CPMG}$ column denotes the time averaged dynamical decoupling decoherence time at an optimal gate number. The quality factor is calculated using $Q = \omega_q T_1$ where ω_q is the qubit frequency.

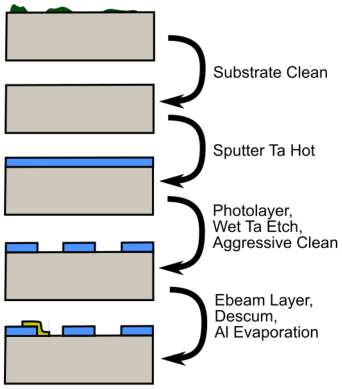

Supplementary Figure 1. Qubit fabrication process. The sapphire substrate (gray) is initially contaminated with carbon (green) which is reduced through substrate cleaning. Tantalum (blue) is then deposited and subsequently patterned with a wet etch. Finally, the Josephson junctions (yellow) are lithographically defined and deposited.

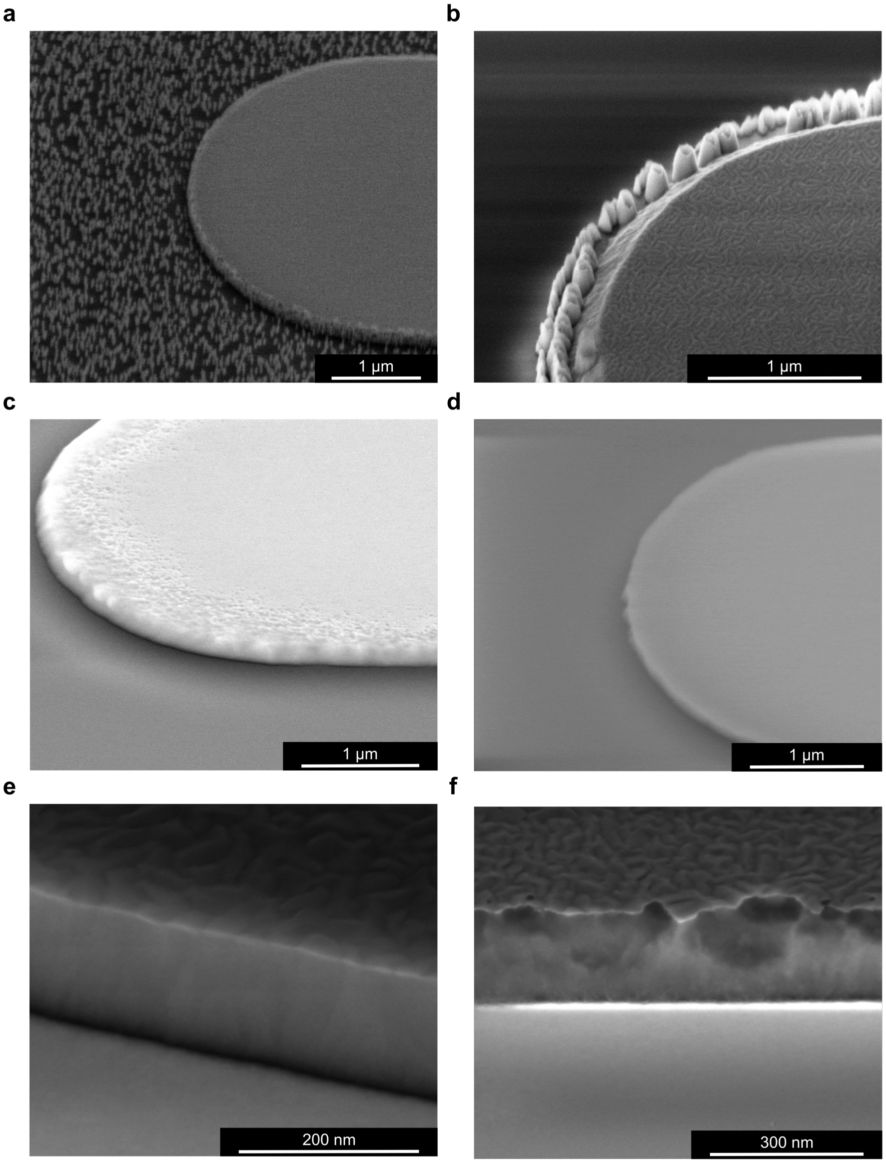

Supplementary Figure 2. Scanning electron microscopy images of tantalum etch development. All panels show etched tantalum on top of sapphire. **a**, **b**, Examples of surface roughening after a 8:3:2 CHF_3:SF_6:Ar dry etch with a 5-7 mTorr pressure and RF/ICP power 30 W/30 W (**a**) and 100 W/100 W (**b**). The rough features near the sidewalls in **b** survived both a piranha clean and an oxygen plasma etch. **c**, Initial wet etch results showed roughening of the tantalum near the edge of the pad, which was circumvented (**d**) by employing a thicker photoresist. **e**, **f**, Higher magnification images of sidewalls etched with the optimized wet etch.

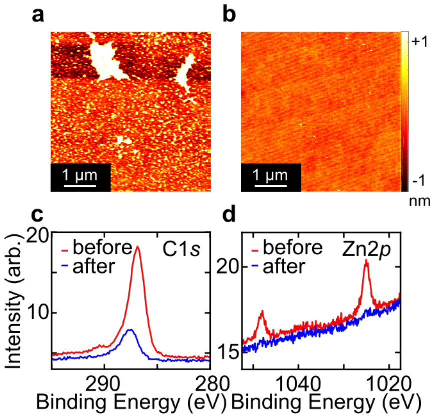

Supplementary Figure 3. Characterization of sapphire surface. AFM images of sapphire after dicing, stripping resist, and solvent cleaning (**a**) and after subsequent piranha cleaning and etching (**b**), showing the removal of particulates from the surface. Average roughness values (R_a) are 400 and 80 pm for **a** and **b**, respectively. XPS of sapphire identifies carbon (**c**) and zinc (**d**) contaminants on the sapphire surface. After piranha cleaning and etching, carbon is reduced by around a factor of five, and zinc is no longer detected. "Before" corresponds to the surface after dicing and solvent cleaning but before acid procedures, and "after" is following acid cleaning steps.

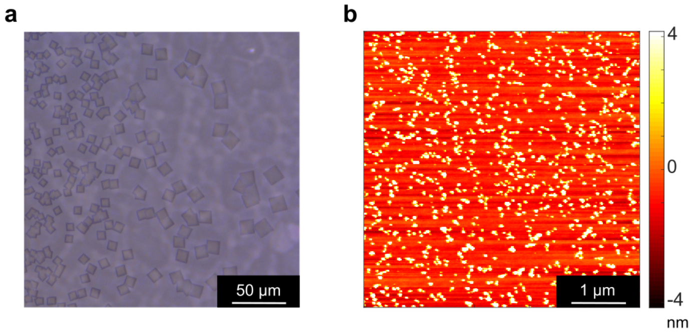

Supplementary Figure 4. Sapphire processing pitfalls. **a**, Optical microscope image of crystals on sapphire after etching in refluxing sulfuric acid for 30 min. **b**, AFM image of sapphire surface showing particulate contaminants after etching and piranha cleaning in borosilicate glassware

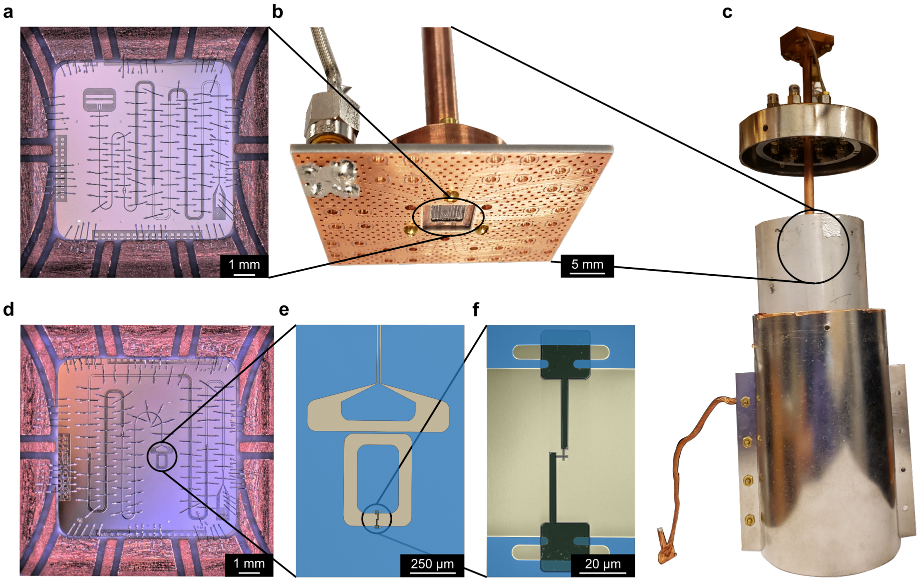

Supplementary Figure 5. Device geometry overview. **a**, Double-pad and (**d**) single-pad geometry transmons mounted to a PCB. In both cases the copper traces of the PCB are visible around the outside of the images, along with the wirebonds providing electrical connections. The excitation and measurement pulses first enter the curving Purcell filter, go through a capacitive coupler to the resonator, then to the qubit. We note in (**d**) that we moved the qubit to the center of the chip. **b**, Device sandwiched between a PCB and aluminum-coated copper which is thermally anchored to the dilution refrigerator. **c**, Exploded view of aluminum (inner) and mu-metal (outer) shields. **e**, Close-up, false-colored SEM image of the single-pad qubit and coupler as well as (**f**) the Josephson junction. The leads from the capacitor pads to the Josephson junction are approximately 2 μm wide.

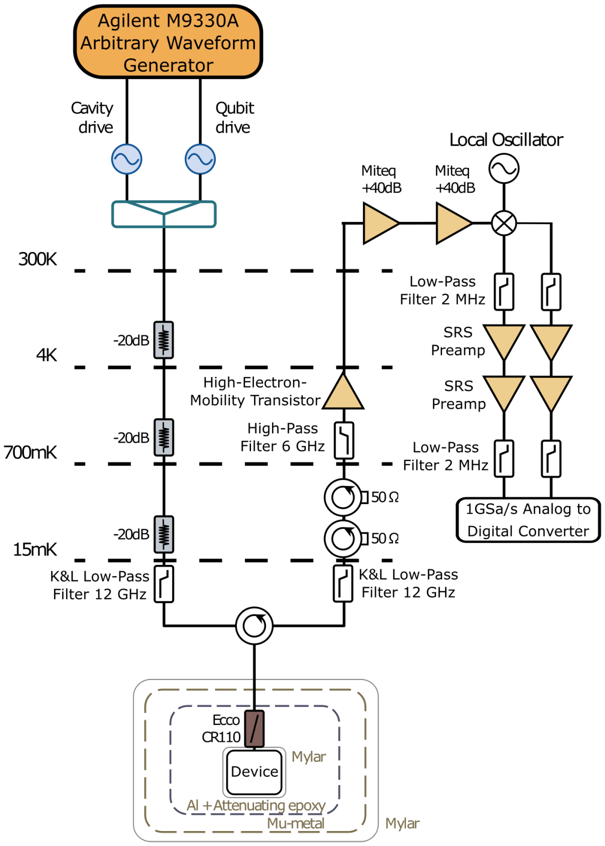

Supplementary Figure 6. Schematic of the measurement electronics and device shielding.

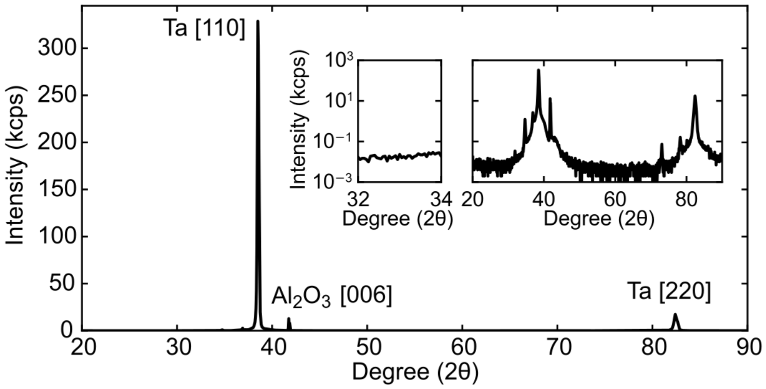

Supplementary Figure 7. X-ray diffraction spectrum of a sputtered tantalum film on sapphire. XRD spectrum of sputtered tantalum on sapphire shows clear peaks corresponding to α-tantalum and sapphire. Inset left, small region of scan plotted on a log scale, showing that we do not detect a β-tantalum [002] peak at 33.7° (2θ)[4]. Inset right, entire scan range on a log scale, showing a few unassigned small peaks which could be caused by contamination, instrumental artifacts, or impurities or defects in the films.

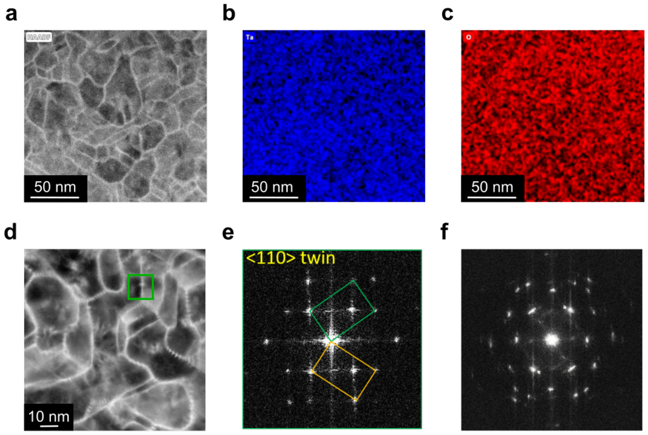

Supplementary Figure 8. Grain boundary characterization. **a**, Plane-view STEM image showing grain boundaries. **b**, **c**, EDS images of the same region shown in **a**, displaying a uniform distribution of tantalum (**b**) and oxygen (**c**). **d**, Atomic resolution STEM image of the boundaries. **e**, Fourier transform of the STEM image at a grain boundary indicated by the green box region of **d**, showing a pattern consistent with twinning. **f**, Fourier transform of the entire image in **d** shows the rotational symmetries of the grains.

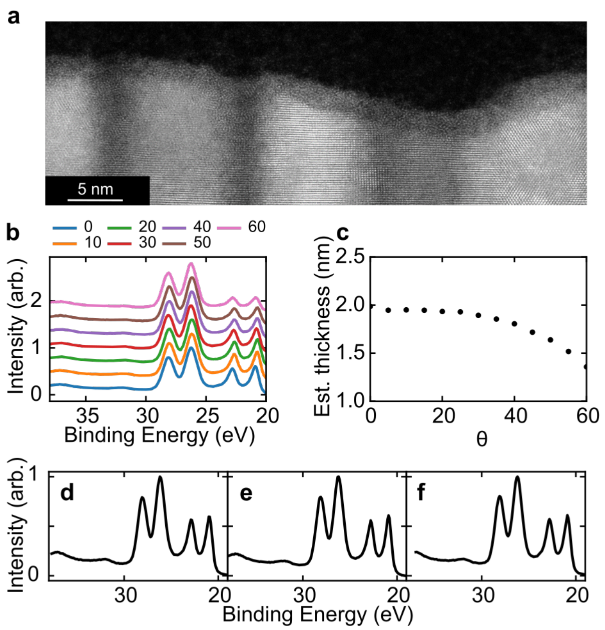

Supplementary Figure 9. Oxide characterization. **a**, Atomic resolution STEM image showing an amorphous oxide layer about 2-3 nm thick on the tantalum surface. **b**, Angle-resolved XPS measurements of Ta4f region of a fabricated device, offset vertically for clarity. Colors indicate the angle in degrees between sample and detector. **c**, Estimated oxide thickness as a function of angle between sample and detector. **d-f**, Ta4f normal incidence XPS data of three completed devices showing nearly identical spectra. The devices were from different tantalum depositions and underwent different fabrication steps. In addition to other variations in fabrication, the device in **d** was only solvent cleaned while the devices surveyed in **e** and **f** were piranha cleaned.

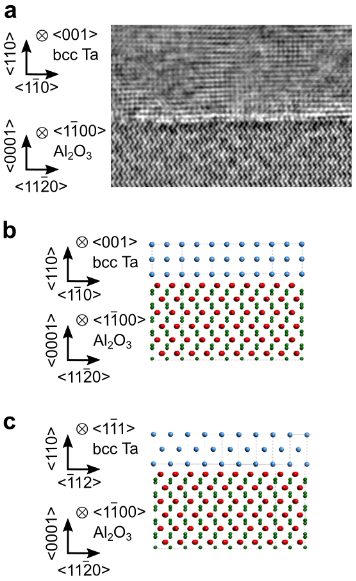

Supplementary Figure 10. Tantalum-sapphire interface characterization. **a**, Atomic resolution iDPC STEM image showing the interface between tantalum and sapphire with the image plane perpendicular to the $\langle 100 \rangle$ direction of tantalum. **b**, Atomistic model of the ideal interface for the tantalum column orientation shown in **a**. **c**, Atomistic model of the ideal interface for the tantalum column orientation shown in Fig. 3e. In both cases oxygen atoms are depicted in red, aluminum in green, and tantalum in blue.

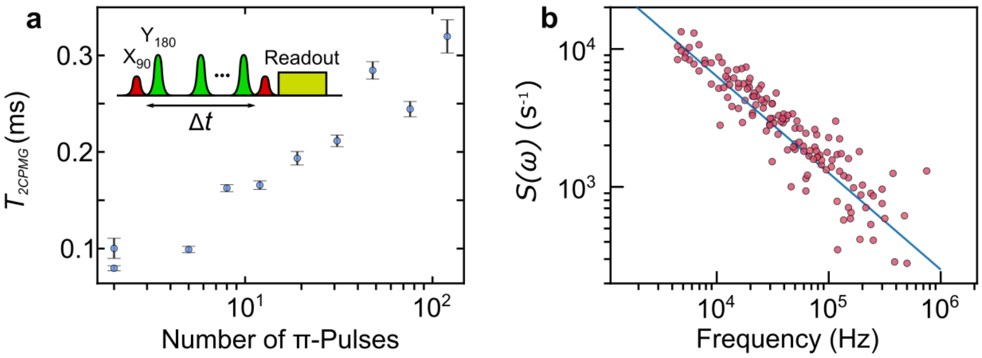

Supplementary Figure 11. Spectral decomposition for Device 11c. **a**, $T_{2,CPMG}$ as an increasing number of pulses reduce the qubit's sensitivity to low-frequency noise. At each point we apply the pulse sequence shown in the inset with a fixed number of π-pulses and vary the delay, Δt, with values ranging from 16 μs to 2 ms. Error bars give the standard deviation in the $T_{2,CPMG}$ fit parameter. Inset: Measurement pulse sequence. X and Y identifies the axis of rotation. Subscripts 90 and 180 refer to a π/2 and π pulse, respectively. **b**, Noise power spectral density S(ω) of the same data as (**a**), following[11]. The blue dashed line indicates a fit by eye to $A/f^{\alpha} + B$ where α = 0.7, A = 2e6s^{-1}, and B = 3e2s^{-1}.

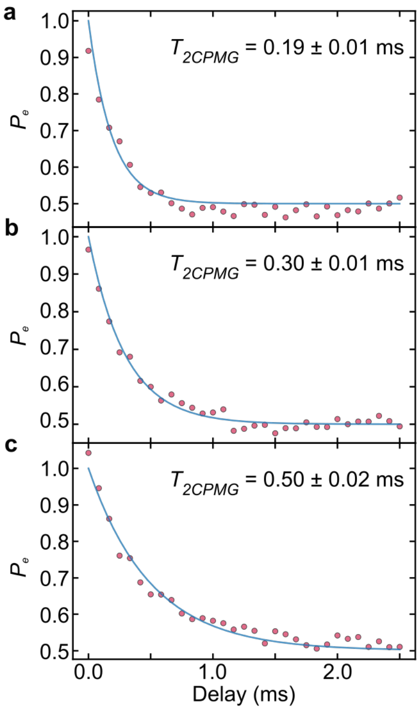

Supplementary Figure 12. CPMG traces. Low (**a**), middle (**b**), and high (**c**) $T_{2,CPMG}$ traces from the data in Fig. 2a, showing the excited state population P_e as a function of delay time. All three traces were fit to a stretched exponential with the exponent constrained to be larger than one.

Supplemental Information References:

1. Barends, R. *et al.* Coherent Josephson qubit suitable for scalable quantum integrated circuits. *Physical Review Letters* **111,** 080502 (2013).
2. Bronn, N. T. *et al.* Broadband filters for abatement of spontaneous emission in circuit quantum electrodynamics. *Applied Physics Letters* **107,** 172601 (2015).
3. Wang, C. *et al.* Surface participation and dielectric loss in superconducting qubits. *Applied Physics Letters* **107,** 162601 (2015).
4. Gladczuk, L., Patel, A., Paur, C. S. & Sosnowski, M. Tantalum films for protective coatings of steel. *Thin Solid Films* **467,** 150–157 (2004).
5. Dwikusuma, F., Saulys, D. & Kuech, T. Study on sapphire surface preparation for III-nitride heteroepitaxial growth by chemical treatments. *Journal of The Electrochemical Society* **149,** G603–G608 (2002).
6. Himpsel, F., Morar, J., McFeely, F., Pollak, R. & Hollinger, G. Core-level shifts and oxidation states of Ta and W: Electron spectroscopy for chemical analysis applied to surfaces. *Physical Review B* **30,** 7236 (1984).

7. McGuire, G., Schweitzer, G. K. & Carlson, T. A. Core electron binding energies in some Group IIIA, VB, and VIB compounds. *Inorganic Chemistry* **12,** 2450–2453 (1973).

8. Moulder, J. F. Handbook of X-ray photoelectron spectroscopy. *Physical Electronics,* 170–171 (1995).

9. Shinotsuka, H., Tanuma, S., Powell, C. & Penn, D. Calculations of electron inelastic mean free paths. X. Data for 41 elemental solids over the 50 eV to 200 keV range with the relativistic full Penn algorithm. *Surface and Interface Analysis* **47,** 871–888 (2015).

10. Cumpson, P. J. Angle-resolved XPS and AES: depth-resolution limits and a general comparison of properties of depth-profile reconstruction methods. *Journal of Electron Spectroscopy and Related Phenomena* **73,** 25–52 (1995).

11. Bylander, J. *et al.* Noise spectroscopy through dynamical decoupling with a superconducting flux qubit. *Nature Physics* **7,** 565–570 (2011).
